# Awareness and Perception Toward Alzheimer’s Disease Among Residents Living in the Jazan Province, Saudi Arabia: A Cross-Sectional Study

**DOI:** 10.7759/cureus.44505

**Published:** 2023-09-01

**Authors:** Faisal Hakami, Mohammed Ali Madkhali, Eman Saleh, Raum Ayoub, Sarah Moafa, Akram Moafa, Bushra Alnami, Bushra Maashi, Saad Khubrani, Wafa Busayli, Abdulaziz Alhazmi

**Affiliations:** 1 Medicine, Faculty of Medicine, Jazan University, Jazan, SAU; 2 Internal Medicine, Hematology and Oncology, Faculty of Medicine, Jazan University, Jazan, SAU; 3 Neuropsychiatry, Faculty of Medicine, Menoufia University, Shibin El Kom, EGY; 4 Microbiology and Pathology, Faculty of Medicine, Jazan University, Jazan, SAU

**Keywords:** jazan, awareness, dementia, ad, alzheimer’s disease

## Abstract

Background: Alzheimer's disease (AD) is a growing public health concern, yet misconceptions about the condition are common. This study assessed awareness and social perceptions of AD in Jazan.

Methods: A cross-sectional survey of 925 adults was conducted. Knowledge was assessed using a 30-item Alzheimer's Disease Knowledge Scale (ADKS). Social perceptions were evaluated using a 10-item questionnaire.

Results: Many had misconceptions about AD epidemiology, causes, management, and care. The mean ADKS score was 8.89 ± 5.17 out of 30. Knowledge was poorest for symptoms, risk factors, treatment, caregiving, and life impact. Knowledge was highest in those aged >45 years (p = 0.018), in those with income > 15K SR (p = 0.004), in retired individuals (p = 0.023), and in those who learned about AD from books (p = 0.001), healthcare professionals (p = 0.001), or had an affected relative (p = 0.001). However, knowledge was low across all domains, averaging only 29% correct answers. Most respondents held positive social perceptions, yet sizable minorities saw isolation, legal intervention, and institutionalization as appropriate. Additionally, a portion of respondents associated stigma with individuals affected by AD and expressed a sense of burden associated with the condition.

Conclusions: There are substantial knowledge gaps and some stigmatizing attitudes about AD in Jazan. Awareness regarding the causes, diagnosis, and management of AD was low. Misconceptions exist that AD only affects older people. Improved public education, especially for higher-risk groups, is needed to address misconceptions and promote social inclusion for those with dementia. Healthcare professionals can play a crucial role.

## Introduction

Alzheimer's disease (AD), a progressive neurodegenerative disorder, is a significant health concern globally due to its escalating prevalence and the associated societal, economic, and healthcare challenges [1,2]. It is marked by an insidious onset and gradual escalation of symptoms such as memory loss, cognitive decline, language difficulty, and behavioral changes [2,3].

AD is the leading cause of dementia, a generic term encompassing various conditions characterized by cognitive impairment [4,5], and is responsible for an estimated 60%-80% of dementia cases worldwide. As of 2021, around 50 million people will have dementia globally. This number is anticipated to triple by 2050 due to the aging world population [6,7].

Despite AD's growing prevalence and severe impact on patients, caregivers, and society, awareness and understanding of the disease remain limited in several communities [8]. This gap can lead to delayed diagnoses, increased stigma, diminished patient quality of life, and heightened caregiver stress [8,9]. Moreover, it can hinder the effectiveness of public health interventions to manage AD and its impacts [9].

Like many other nations, Saudi Arabia is undergoing significant demographic transformations due to increased life expectancy and a growing elderly population [10,11]. These changes are expected to raise the prevalence of age-related diseases, including AD. Several studies highlight the escalating prevalence of dementia in Saudi Arabia, emphasizing the need for effective strategies to manage this expanding health challenge [12,13]. The country's dementia prevalence is around 6.4% [13]. However, previous studies reveal that many individuals and their families perceive AD symptoms as normal aging, leading to a delay in seeking medical treatments [13]. Managing AD's severity is a complex process due to overlapping symptoms of cognitive decline and behavioral changes following pharmacological treatment, necessitating widespread community awareness and knowledge [14].

Previous research indicates average dementia knowledge among healthcare students, with a noticeable improvement from the first to the final year of study [15,16]. Several studies estimating AD knowledge among the general public have been published [17,18]. Jeddah-based research found that 89% of 1511 people had heard of AD. Approximately 46% of participants believed a brain disease caused AD, while 44.9% considered it a normal part of aging [17]. The average knowledge score in the Aseer study with 1374 participants was 10.77 ± 5.11. Younger participants and those with a family history of the disease demonstrated better AD knowledge [18].

The Jazan Province, located in southwestern Saudi Arabia, is likely to experience the impact of the rising prevalence of AD. However, the current level of AD awareness and perception among its residents remains largely unknown. Understanding these perspectives is vital as public awareness and perception influence health-seeking behaviors, caregiver support, and community attitudes toward dementia.

Public awareness about AD can boost early detection and diagnosis, which is vital for disease management. It can also affect an individual's willingness to seek medical advice when symptoms emerge and their acceptance of support services [19]. Perceptions of AD can shape societal attitudes toward people with dementia, influencing their quality of life and social inclusion. Negative perceptions can lead to stigma, social isolation, and discrimination, while positive perceptions can nurture empathy, social support, and improved care for AD patients [20].

Given these considerations, this study aims to evaluate the awareness and perceptions of AD disease among Jazan Province, Saudi Arabia residents. The study employs a cross-sectional design, surveying a representative population sample to ascertain their knowledge about AD, their attitudes toward the disease, and those affected.

## Materials and methods

Study design and population

This is an observational cross-sectional study of adults who are above the age of 18 and are currently residing in the Jazan region. Additionally, participants who possessed awareness or had heard of the disease were included, while those not meeting these criteria were excluded from the study. The study aims to evaluate public awareness of AD and their social attitudes using an online survey through a convenience sampling method.

Sampling procedures

To conduct this study, a minimum of 384 participants were required. The sample size was determined using the formula for a single cross-sectional survey:

$n=\frac{[Z^{2}pq]}{d^{2}}$

In this case, the parameters used were as follows: p represented the assumed awareness level at 50%, Z indicated a 95% confidence interval, q was equal to 1-p, and d represented an acceptable error margin of no more than 4%. To mitigate potential biases in the sample, we tripled its size to encompass approximately 900 participants.

Data collection, study instrument, and scoring system

A self-administered online questionnaire designed using Google platforms was distributed to the participants for assessment of the awareness and perception toward AD among residents living in Jazan Province, Saudi Arabia. The constructed questionnaire included many sections to achieve the purpose of the study. The first section collected participant demographics such as age, gender, education, nationality, residency, occupation, income, and social habits such as smoking, and experience of Alzheimer's in his/her family member. The second section is to assess the general information about AD and social attitudes regarding Alzheimer’s patients, the participant’s attitude, his/her concerns and beliefs about AD, and how he/she is dealing with AD patients. The third section is focused mainly on assessing the participants’ knowledge of AD via the Alzheimer’s Disease Knowledge Scale (ADKS). This section is further subdivided into seven subdivisions including true-or-false questions about Alzheimer's risk factors, assessment and diagnosis, symptoms, course, life impact, care provided, and treatment and management. The questionnaire was designed in English and then translated into Arabic by professional translators.

Ethical approval and informed consent

Ethical approval was obtained from the Jazan University Ethical Committee (Reference Number: REC-44/07/534). Informed consent was obtained from all participants, and the privacy rights and confidentiality of the participants were maintained.

Data analysis

The statistical analyses were conducted utilizing SPSS, version 25 (IBM Corp., Armonk, NY). Descriptive statistics were computed for all variables in the form of frequencies and percentages for categorical data and mean and standard deviation (SD) for continuous data. Non-parametric tests such as the Mann-Whitney U test for variables with two categories and the Kruskal-Wallis test for variables with three or more categories were employed.

## Results

A total of 925 participants completed the survey and were included in the study. The mean age of the participants was 30.58 ± 10.34 years. Most participants, 47.6%, were 18 to 25 years old. More than half (57.1%) of the participants were male. About 55.7% of the participants lived in rural areas and 44.3% in urban areas. Regarding the education level, 25.4% had completed high school or had less education, 71.9% had bachelor's degrees, and 2.7% had master's or Ph.D. degrees. Almost equal percentages of participants were married (43.7%) and unmarried (56.3%). Monthly income was less than 5K for 47.2%, between 5K and 15K for 39.4%, and more than 15K for 13.4% of the participants. In occupation, 40.2% were university/school students and government employees each, 6.8% had a private job, 3.8% were retired, and 9% were unemployed. Regarding social habits, 10.2% consumed Qat, 15% were smokers, and 1.9% used non-smoking tobacco. The primary sources of information about AD were social media, the internet, TV (53.3%), relatives or friends (36.4%), educational institutions (30.5%), books (19.7%), healthcare professionals (21.9%), personal experience (22.5%), and others (10.6%). About 22.7% of the participants had a relative with AD (Table 1).

**Table 1:** Sociodemographic characteristics of the study subjects (n = 925) *Study participants who answered that they did not know about Alzheimer's disease yet were excluded from our study.

Characteristics	
Age (Years)	Mean ± SD/n (%)
Mean ± SD	30.58 ± 10.34
18-25	440 (47.6%)
26-35	213 (23%)
36-45	167 (18.1%)
>45	105 (11.4%)
Gender	
Male	528 (57.1%)
Female	397 (42.9%)
Type of residency	
Rural	515 (55.7%)
Urban	410 (44.3%)
Educational level	
High school/diploma or less	235 (25.4%)
Bachelor’s degrees	665 (71.9%)
Master/Ph.D. degrees	25 (2.7%)
Marital status	
Married	404 (43.7%)
Not married	521 (56.3%)
Monthly income	
Less than 5K	437 (47.2%)
More than 5K, but less than 15K	364 (39.4%)
More than 15K	124 (13.4%)
Occupation	
University/School student	372 (40.2%)
Government employee	372 (40.2%)
Private employee	63 (6.8%)
Retired/Others	35 (3.8%)
Unemployed	83 (9%)
Social habits	
Qat consumption	94 (10.2%)
Smoking	139 (15%)
Smokeless tobacco	18 (1.9%)
Source of information you received about Alzheimer’s disease*	
Social media/Internet websites/TV	493 (53.3%)
Relatives or friends	337 (36.4%)
Educational institution	282 (30.5%)
Books	182 (19.7%)
Healthcare professionals	203 (21.9%)
Personal experience	208 (22.5%)
Others	98 (10.6%)
Do you have a relative with Alzheimer's disease?	
No	715 (77.3%)
Yes	210 (22.7%)

The general information regarding AD is presented in Table 2. When asked whether AD is restricted to a specific age group, 73.6% thought that it only affects older people, 0.3% thought that it only affects younger people, 0.4% thought that it only affects children, 14.6% said that it could occur at any age, and 11% did not know. Regarding restriction to a specific gender, 9% thought it only affects males, 5.3% thought it only affects females, 72.1% said it could occur regardless of gender, and 13.6% did not know.

**Table 2:** General information regarding Alzheimer’s disease *Participants were given the opportunity to select multiple answers for this question rather than being limited to choosing only one response.

Question	
Do you think Alzheimer's disease is restricted to a specified age group?	
Yes, to elderlies	681 (73.6%)
Yes, to youngsters	3 (0.3%)
Yes, to children	4 (0.4%)
No (it can occur at any age)	135 (14.6%)
I don’t know	102 (11%)
Do you think Alzheimer's disease is restricted to a specific sex?	
Yes, to males	83 (9%)
Yes, to females	49 (5.3%)
No (It can occur regardless of sex)	667 (72.1%)
I don’t know	126 (13.6%)
What are the causes of Alzheimer’s disease?*	
God’s punishment	17 (1.8%)
Evil eye or magic	23 (2.5%)
Brain disease (organic or psychotic)	385 (41.9%)
Genetic causes	177 (19.1%)
Head injury	126 (13.6%)
No specific cause	499 (53.9%)
Other causes	28 (3%)
I don’t know	194 (21%)
Do you think that Alzheimer’s disease is controlled?	
Yes, it’s controlled through*	310 (33.5%)
Medically	273 (29.5%)
Surgically	33 (3.6%)
Electrical shock	15 (1.6%)
Reading Quran	89 (9.6%)
Other options	16 (1.7%)
No, it’s not treatable	233 (25.2%)
I don’t know	382 (41.3%)

The perceived causes of AD were the following: God's punishment (1.8%), evil eye or magic (2.5%), brain disease (41.9%), genetic causes (19.1%), head injury (13.6%), there is no specific cause (53.9%), other reasons (3%), and I don't know (21%).

Nearly one-third of the participants (33.5%) believe that AD is treatable, while 25.2% thought it was not treatable, and 41.3% did not know. Regarding treatment options, 29.5% thought it could be treated medically, 3.6% thought surgically, 1.6% thought through electrical shock, 9.6% thought through reading the Quran, and 1.7% thought through other modalities of therapy.

A significant limitation is the low level of awareness about the age distribution, causes, and management of AD as better visualization for the presenting data in Table 2.

Knowledge of AD was assessed using a 30-item AD knowledge scale (Table 3). For risk factors, 49.4% correctly identified that mental exercise could help prevent AD, 31.5% knew that people in their 30s can have the condition, 18% knew that high cholesterol increases the risk, 27.4% knew that prescription drugs slow progression are available, 22.4% knew that high blood pressure increases the risk, and 43.6% knew that genes are only partially responsible.

**Table 3:** Alzheimer’s Disease Knowledge Score scale *Correct answer.

Item	True	False	I don’t know
Risk factors			
1. Mental exercise has been scientifically proven to prevent a person from getting Alzheimer’s disease.	457 (49.4%)	63 (6.8%)*	405 (43.8%)
2. People in their 30s can have Alzheimer’s disease.	291 (31.5%)*	165 (17.8%)	469 (50.7%)
3. Having high cholesterol can increase a person’s risk of developing Alzheimer's disease.	166 (18%)*	84 (9%)	675 (73%)
4. Prescription drugs that help delay the progression of Alzheimer’s disease are available.	253 (27.4%)	137 (14.8%)*	535 (57.8%)
5. High blood pressure may increase the risk of developing Alzheimer’s disease.	207 (22.4%)*	103 (11.1)	615 (66.5%)
6. Genes can only partially account for the development of Alzheimer’s disease.	403 (43.6%)*	72 (7.8%)	450 (48.6%)
Life impact			
7. People with Alzheimer’s disease are particularly prone to depression.	461 (49.8%)*	108 (11.7%)	356 (38.5%)
8. Most people with Alzheimer’s disease live in nursing homes.	166 (17.9%)	337 (36.4%)*	422 (45.6%)
9. It is safe for people with Alzheimer’s disease to drive as long as they have a companion in the car at all times.	706 (76.8%)	23 (2.5%)*	196 (21.2%)
Assessment and diagnosis			
10. When a person with Alzheimer’s disease is agitated, a medical assessment can reveal other health problems that caused the agitation.	296 (32%)*	54 (5.8%)	575 (62.2%)
11. If memory and confusion start suddenly, it is likely due to Alzheimer’s disease.	333 (36%)	168 (18.2%)*	424 (45.8%)
12. Symptoms of severe depression can be mistaken for symptoms of Alzheimer’s disease.	185 (20%)*	248 (26.8%)	492 (53.2%)
13. Alzheimer’s disease is one type of dementia.	581 (62.8%)*	87 (9.4%)	257 (27.8%)
Course			
14. After the symptoms of Alzheimer’s disease appear, the average life expectancy is 6 to 12 years.	96 (10.4%)*	131 (14.2%)	698 (75.5%)
15. In rare cases, people have recovered from Alzheimer’s disease.	275 (29.7%)	97 (10.5%)*	553 (59.8%)
16. An individual with Alzheimer’s is likelier to fall as the disease worsens.	549 (59.4%)*	28 (3%)	348 (37.6%)
17. Eventually, a person with Alzheimer’s will need 24-hour supervision.	523 (56.5%)*	142 (15.4%)	260 (28.1%)
Care giving			
18. People with Alzheimer’s disease do best with simple instructions given one step at a time.	504 (54.5%)*	58 (6.3%)	363 (39.2%)
19. When people with Alzheimer’s disease have difficulty caring for themselves, caregivers should take over immediately.	675 (73%)	75 (8.1%)*	175 (18.9%)
20. If a person with Alzheimer’s disease becomes alert and agitated at night, a good strategy is to ensure that the person gets plenty of physical activity during the day.	284 (30.7%)*	50 (5.4%)	591 (63.9%)
21. When people with Alzheimer’s disease repeat the same question or story several times, it is helpful to remind them that they are repeating themselves.	218 (23.6%)	425 (45.9%)*	282 (30.5%)
22. Once people have Alzheimer’s disease, they can no longer make informed decisions about their care.	491 (49.8%)	134 (14.5%)*	330 (35.7%)
Treatment			
23. People whose Alzheimer’s disease is not yet severe can benefit from psychotherapy for depression and anxiety.	410 (44.3%)*	49 (5.3%)	466 (50.4%)
24. Poor nutrition can worsen the symptoms of Alzheimer's disease.	440 (47.6%)*	83 (9%)	402 (43.5%)
25. When a person has Alzheimer’s disease, using reminder notes can contribute to the decline.	548 (59.2%)	64 (6.9%)*	313 (33.8%)
26. Alzheimer’s disease cannot be cured.	199 (21.5%)*	140 (15.1%)	586 (63.4%)
Symptoms			
27. Tremor or shaking of the hands or arms is a common symptom in people with Alzheimer’s disease.	230 (24.9%)	189 (20.4%)*	506 (54.7%)
28. Trouble managing money or paying bills is a common early symptom of Alzheimer’s disease.	239 (25.8%)*	242 (26.2%)	444 (48%)
29. One symptom that can occur with Alzheimer’s disease is believing that other people are stealing things.	335 (36.2%)*	139 (15%)	451 (48.8%)
30. Most people with Alzheimer’s disease remember recent events better than things that happened in the past.	222 (24%)	303 (32.8%)*	400 (43.2%)

For life impact, 49.8% knew that depression is common, 17.9% wrongly thought that most people live in nursing homes, and 76.8% wrongly believed that driving with a companion is safe for people with AD. For evaluation and diagnosis, 32% knew that agitation could indicate other health problems, 36% wrongly thought that sudden memory trouble is likely due to AD, 20% knew that depression symptoms could resemble AD, and 62.8% knew that AD is a type of dementia.

For the course, 10.4% correctly identified the average life expectancy after symptoms as 6-12 years, 29.7% incorrectly thought that recovery is possible, 59.4% knew that falls become more likely as the disease progresses, and 56.5% knew that 24-hour supervision is needed. For caregiving, 54.5% knew that simple step-by-step instructions are best, 73% knew that caregivers should take over when self-care becomes difficult, 30.7% knew that daytime activity helps reduce nighttime agitation and restlessness, 23.6% wrongly thought that reminding patients that they are repeating themselves is helpful, and 49.8% wrongly thought that patients lose decision-making capacity.

For treatment, 44.3% knew that early-stage patients could benefit from psychotherapy, 47.6% knew that good nutrition is important, 59.2% knew that using reminder notes is harmful, and 21.5% incorrectly thought there is no cure. For symptoms, 24.9% incorrectly identified the tremor as common, 25.8% knew that trouble managing money is common, 36.2% knew that believing others are stealing is common, but 24% wrongly thought that recent memory is better.

**Figure 1:**
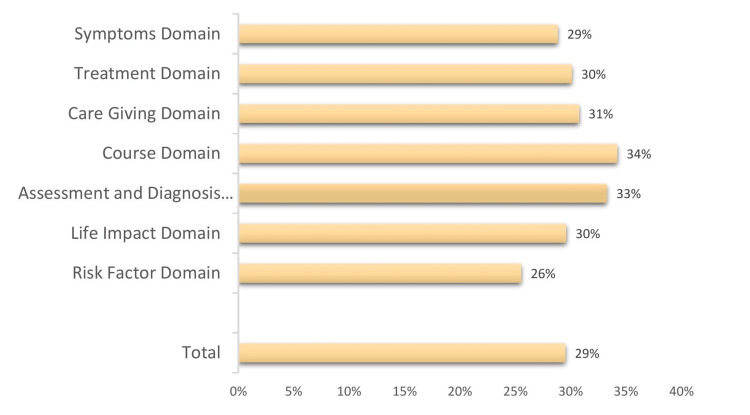
ADKS domains' correct response rates ADKS: Alzheimer's Disease Knowledge Scale.

Figure 1 shows that the domain with the highest percentage of correct answers was the course domain, with 34% of the people correctly answering questions related to the progression of the disease. The assessment and diagnosis domain followed closely behind, with 33% of people correctly answering questions related to the diagnosis process.

However, the remaining domains had significantly lower percentages of correct answers. The symptoms domain, with only 29% of people answering questions related to the symptoms of AD correctly. The treatment, caregiving, life impact, and risk factor domains also had low percentages of correct answers, with 30%, 31%, 30%, and 26%, respectively. The correct answers from the total domains were only 29%.

Knowledge scores were analyzed based on demographic characteristics (Table 4). The mean ADKS score was 8.89 ± 5.17. There were significant differences in mean scores based on age (p = 0.018), income (p = 0.004), occupation (p = 0.023), learning about AD from educational institutions (p < 0.001), books (p < 0.001), healthcare professionals (p = 0.001), personal experience (p = 0.019) and having a relative with the disease (p < 0.001).

**Table 4:** Demographic characteristics and mean score knowledge *Significant value (0.05 or less) in the Mann-Whitney U or Kruskal-Wallis tests. ADKS: Alzheimer's Disease Knowledge Scale.

Characteristics	ADKS (Mean ± SD)	P-value
Age (Years)		
18-25	8.92 ± 5.10	
26-35	8.80 ± 5.38	0.018*
36-45	8.05 ± 5.15	
>45	9.90 ± 4.63	
Gender		
Male	8.59 ± 5.05	0.089
Female	9.19 ± 5.25	
Type of residency		
Rural	8.75 ± 5.27	0.48
Urban	8.97 ± 4.97	
Educational level		
High school/Diploma or less	8.42 ± 5.23	
Bachelor’s degrees	8.93 ± 5.11	0.055
Master/Ph.D. degrees	10.68 ± 4.91	
Marital status		
Married	8.78 ± 5.02	0.746
Not married	8.90 ± 5.24	
Monthly income		
Less than 5K	8.83 ± 5.19	
More than 5K, but less than 15K	8.41 ± 5.01	0.004*
More than 15K	10.17 ± 5.16	
Occupation		
University student	9.38 ± 5.12	
Government employee	8.28 ± 5.11	
Private employee	8.78 ± 5.08	0.023*
Retired/others	9.80 ± 5.02	
Unemployed	8.66 ± 5.29	
Qat consumption		
No	8.92 ± 5.16	0.173
Yes	8.20 ± 4.94	
Smoking		
No	8.94 ± 5.21	0.170
Yes	8.33 ± 4.70	
Smokeless tobacco		
No	8.86 ± 5.17	0.724
Yes	8.44 ± 3.58	
I know the disease from social media/internet websites/TV		
No	8.79 ± 5.37	0.901
Yes	8.89 ± 4.94	
I know the disease from relatives or friends		
No	8.90 ± 5.19	0.797
Yes	8.76 ± 5.06	
I know the disease from educational institution		
No	8.30 ± 5.15	<0.001*
Yes	10.41 ± 5.02	
I know the disease from books		
No	8.46 ± 5.10	<0.001*
Yes	10.41 ± 5.02	
I know the disease from healthcare professionals		
No	8.55 ± 5.12	0.001*
Yes	9.92 ± 5.08	
I know the disease from personal experience		
No	8.62 ± 5.02	0.019*
Yes	9.62 ± 5.50	
Do you have a relative with Alzheimer's disease?		
No	8.38 ± 5.10	<0.001*
Yes	10.45 ± 4.98	

Knowledge was highest for those who are aged > 45 years (9.90 ± 4.63), those with a monthly income >15K (10.17 ± 5.16), those who are students (9.38 ± 5.12), retired (9.80 ± 5.02), those who are learning from educational institutions (10.41 ± 5.02), books (10.41 ± 5.02), healthcare professionals (9.92 ± 5.08), personal experience (9.62 ± 5.50), and those with an affected relative (10.45 ± 4.98). Knowledge was lowest for those who are aged 36-45 years (8.05 ± 5.15), those who have monthly income of 5-15K (8.41 ± 5.01), those who are government employees (8.28 ± 5.11), and those without the indicated sources of information or experience.

There were no significant differences in knowledge based on gender, residency, education, marital status, qat use, smoking, smokeless tobacco use, social media, friends/relatives, or occupation, except retired/others (Table 4).

Social perceptions of AD were assessed using 10 questions (Table 5). The majority did not believe it is advisable to isolate patients from activities to prevent embarrassment (74.1%), did not think legal intervention is essential when challenges arise (67.2%), did not feel embarrassed by a relative's diagnosis (81.2%), did not tend to reject a relative's diagnosis (84.3%), did not support nursing home over in-home care (84.6%), thought public outings are feasible (73.7%), did not think patients are stigmatized (79.1%), did not see them as a burden (81%), and did not think they should be isolated (52.9%).

**Table 5:** Social perception of Alzheimer's disease

Question	Yes	No
Do you believe it is advisable to keep the patient diagnosed with Alzheimer's from attending social gatherings and engaging in daily activities to prevent the patient from feeling embarrassed?	240 (25.9%)	685 (74.1%)
If a patient with Alzheimer's is experiencing challenges in carrying out routine tasks, do you believe it is essential to seek legal intervention to protect the patient's rights?	303 (32.8%)	622 (67.2%)
Do you experience feelings of embarrassment if a family member is diagnosed with Alzheimer's disease?	174 (18.8%)	751 (81.2%)
Do you tend to reject the diagnosis when a family member is diagnosed with Alzheimer's?	145 (15.7%)	780 (84.3%)
Do you support having Alzheimer's patients receive care in state-run nursing homes rather than being cared for at home?	142 (15.4%)	783 (84.6%)
Is it feasible to transport people with Alzheimer's disease to a public location?	682 (73.7%)	243 (26.3%)
Do you believe that people with Alzheimer's disease are stigmatized by society?	193 (20.9%)	732 (79.1%)
Do you think individuals with Alzheimer's disease pose a burden on their families?	176 (19%)	749 (81%)
Do you believe that people with Alzheimer's disease should be isolated from society?	436 (47.1%)	489 (52.9%)

However, 25.9% believed isolation to be beneficial, 32.8% saw legal intervention as essential, 18.8% felt embarrassed, 15.7% would reject the diagnosis, 15.4% preferred nursing homes, 26.3% saw public trips as infeasible, 20.9% saw stigma, 19% saw patients as a burden, and 47.1% supported isolation (Table 5).

## Discussion

This survey of 925 participants in the Jazan region of Saudi Arabia found significant gaps in knowledge and some stigmatizing perceptions about AD. On average, participants correctly answered only 29% of questions about AD. Knowledge was highest for the AD course (34% correct) but lowest for risk factors (26% correct). This finding aligns with past studies showing poor public awareness and knowledge of AD [18,21].

Demographic factors linked to higher knowledge included older age, higher income, being a student or retired, and learning about AD from educational sources, healthcare professionals, personal experience, or an affected relative. These groups likely have more exposure to information about AD [22-24]. In contrast, lower knowledge among those aged 36-45 years, with middle incomes, or government employees suggests less access to information [22]. Past investigations reported that factors associated with greater knowledge of AD include working in the dementia field, attending a related class or support group, exposure to AD information and experiences, and having family members with AD [22,23]. Knowledge levels were similar regardless of gender, residence, education, marital status, and social habits like qat use or smoking. While informative, demographic factors only partly explained knowledge gaps. Qualitative work could explore other influences on knowledge, like culture or beliefs. Overall, groups with less knowledge represent targets for education on AD symptoms, treatments, caregiving, and living with the disease.

For risk factors, 49.4% of respondents correctly identified that mental exercise could help prevent AD. This indicates a relatively high level of awareness about the potential benefits of cognitive stimulation in maintaining brain health. Mental practices such as puzzles, reading, learning new skills, and engaging in challenging activities have reduced cognitive decline risk [25,26]. In contrast, only 18% of respondents knew that high cholesterol increases the risk of AD. This indicates a significant knowledge gap. Research shows that high cholesterol levels, particularly in midlife, may contribute to developing amyloid plaques and tau tangles, the hallmark brain abnormalities associated with AD [27,28]. Educating the public about the link between cholesterol and AD risk could promote lifestyle changes and better cardiovascular health.

Similarly, only 22.4% of respondents recognized high blood pressure as a risk factor for AD. This lack of understanding of the relationship between hypertension and cognitive health is problematic. Research suggests chronic high blood pressure can damage blood vessels and reduce blood flow to the brain, increasing the risk of cognitive impairment and dementia [29,30]. Improving public knowledge about this connection may encourage individuals to monitor and manage their blood pressure more effectively. It is encouraging that 43.6% of individuals recognized that genes are only partially responsible for AD. While genetic factors can contribute to the risk of developing the condition, they do not solely determine whether someone will develop AD. Environmental factors, lifestyle choices, and other risk factors play significant roles [31,32]. Understanding AD as multifactorial empowers individuals to make informed health decisions and adopt preventive measures. Previous research found even lower awareness of risk factors like hypertension and high cholesterol, with only 15.7% and 14.9% of participants identifying them, respectively [18]. These findings highlight the need to educate the public about AD risk factors and prevention strategies to support healthier aging.

Most participants held supportive views, disagreeing that isolation, embarrassment, rejecting an AD diagnosis, preferring nursing homes, avoiding public outings, stigma, and burden were appropriate. However, some endorsed less supportive views that could worsen difficulties for those with AD and caregivers. Stigmatizing attitudes persist and may relate to a lack of awareness and experience [33]. Studies have found that caring for a person diagnosed with AD hurts family caregivers' psychological health [34,35]. Caregivers' perceptions of the negative aspects of caregiving comprise emotional and social factors, such as fear, worry, stress, sadness, and social isolation [34,36,37]. In the current study, negative perceptions included seeing isolation as beneficial (25.9%), legal intervention as essential (32.8%), feeling embarrassed by a relative’s diagnosis (18.8%), rejecting a relative’s diagnosis (15.7%), preferring nursing homes (15.4%), avoiding public outings (26.3%), stigma (20.9%), and burden (19%). These views could worsen difficulties for those with AD and their caregivers by reducing social support and delaying diagnosis and treatment. Therefore, addressing negative perceptions of AD is essential to improve patients' and caregivers' quality of life [38]. In the present study, most disagreed with stigmatizing views, seeing isolation (74.1%), legal intervention (67.2%), and nursing home preference (84.6%) as unnecessary and public outings as feasible (73.7%). They also did not see AD as stigmatizing (79.1%) or patients as burdensome (81%), showing supportive attitudes. Qualitative work could explore the roots of stigmatizing perceptions to address them through education better. Campaigns promoting stories of living well with AD and caregiving experiences could help build understanding and empathy to counter stigma.

Knowledge gaps and negative perceptions must be addressed through education and campaigns promoting understanding of AD and reducing stigma. Education should manage symptoms, treatments, caregiving, and stigma targeting groups with less knowledge and experience of AD. Personal stories from patients and caregivers may be impactful as knowledge and perceptions were most positive among those learning from these sources or an affected relative [23,33].

Misconceptions were evident, with 73.6% incorrectly believing AD only affects older people and 53.9% believing it always occurs in older people. Early-onset AD can happen in the 30s and 40s [39]. Nearly half (41.3%) did not know about possible treatments, though medications and strategies can slow AD progression and manage symptoms [40]. These misconceptions demonstrate the need for public education on AD.

Limitations of this study include reliance on self-reported data, potential social desirability bias, a cross-sectional design preventing analysis of changes over time, a sample from only one Saudi province, and a lack of qualitative data to explore reasons for knowledge gaps and perceptions. Future research could examine how to tailor education for groups with less knowledge and more negative views of AD. Studies could assess the impacts of campaigns on knowledge, perceptions, and behaviors toward those with AD.

## Conclusions

This study assesses awareness and perceptions of AD in Jazan Province, Saudi Arabia. Significant knowledge gaps and some stigmatizing attitudes were found and must be addressed through education to better support those with AD and caregivers. On average, less than 50% of questions were answered correctly on a 30-item AD knowledge scale. Knowledge was highest for the AD course but lowest for risk factors. Widespread misconceptions were that AD only affects older people and that all elderly will develop it. Nearly half did not know about available treatments. Some groups, including older adults, higher-income individuals, students, retirees, and those learning from educational sources, healthcare professionals, or personal experience, had more knowledge. These groups likely have more exposure to information on AD. Knowledge was lower in middle-aged adults, middle-income groups, and government employees, suggesting less access to information.

Most held supportive views of AD, but some endorsed stigmatizing attitudes that could worsen difficulties for patients and caregivers. Negative perceptions included seeing isolation and legal intervention as beneficial, feeling embarrassed by an AD diagnosis, preferring nursing homes, avoiding public outings, and seeing patients as stigmatized or burdensome. Ongoing efforts are needed to address knowledge gaps and combat stigma related to AD in Saudi Arabia. Gaining a deeper understanding of cultural and social influences on perceptions can help develop targeted education campaigns to overcome the barriers to diagnosis and care. Continued research should assess awareness programs' evolving perceptions and impacts over time. Enhanced support for those with AD today and better care for future generations depend on an informed and compassionate public.
